# The impact of simulation-based learning on the knowledge, attitude and performance of physiotherapy students on practice placement

**DOI:** 10.1186/s12909-024-05718-2

**Published:** 2024-07-22

**Authors:** Yetunde M. Dairo, Kirsty Hunter, Timothy Ishaku

**Affiliations:** 1 Department of Physiotherapy, School of Health and Social Care Profession, Buckinghamshire New University, Queen Alexandra Rd, High Wycombe, HP11 2JZ UK; 2 Division of Surgery and Interventional Science, Institute of Sports, Exercise and Health (ISEH), 170 Tottenham Court Road, London, W1T 7HA UK

**Keywords:** Practice placement, Simulation-based learning, Physiotherapy, Healthcare, Higher-education

## Abstract

**Background:**

Practice placement is a crucial part of pre-registration physiotherapy education worldwide. However, educators face challenges in finding practice sites for students to undertake placement. The use of simulation-based (SBL) learning has the potential to replace some traditional placement, thereby increasing placement capacity, but the benefits have not been fully explored. This study aimed to assess the impact of SBL placement on students’ knowledge, attitude, and performance during practice placements with external providers.

**Methods:**

This study utilised an exploratory qualitative research design using a semi-structured interview to collect data from Year 2 students of a 2-year MSc pre-registration physiotherapy programme in the UK. To be eligible to be included in the study, students must have participated in the 4-week simulation placement and have completed all their practice placements with external providers. All the interviews were conducted virtually in a 1:1 setting and recorded. The interviews lasted an average of 45 min. An inductive thematic analysis based on Braun and Clarke’s approach was utilised in this study.

**Results:**

Twelve students consented to participate in this study. The 56 codes generated from the data were categorised into 5 themes; [1] Working together, [2] Working with Service Users, [3] Professionalism, [4] Profession-specific practical skills and [5] Generic practical skills. Participants unanimously expressed a positive view on working in groups, and they believe that engaging with service users who acted as patients enhanced the authenticity of the simulation placement. Subjective and objective assessments were considered important profession-specific skills gained during the simulation. Despite the benefits derived from the simulation placement, some participants felt that the learning experience would have been enriched by periodically changing the groups they were working in and having the opportunity for more 1:1 feedback throughout the four weeks.

**Conclusions:**

SBL has the potential to be a valuable educational experience for physiotherapy students. It may assist in better preparing students for successful integration into the dynamic healthcare environment. To enhance and improve the authenticity of this type of placementour recommendations include recruiting more service users, incorporating and encouraging more intervention-based elements, and increasing the practice educators’ and students’ contact time.

**Supplementary Information:**

The online version contains supplementary material available at 10.1186/s12909-024-05718-2.

## Introduction and background

Practice placement is a crucial part of pre-registration physiotherapy education worldwide. It allows learners to apply and reinforce their academic learning in a real workplace setting [1, 2]. In the UK, universities partner with hospitals, clinics, and other practice settings to provide a safe and structured placement environment, where learners can achieve pre-defined learning outcomes, and are assessed accordingly [3]. The expected outcome is that learners who have completed a minimum of 1000 h in diverse practice settings as part of their physiotherapy degree will have attained the necessary competency to become registered physiotherapists [2]. However, educators face challenges in finding practice sites to prepare physiotherapy students, including a shortage of qualified staff and practice educators, and an increase in the number of learners and universities offering physiotherapy programmes without a corresponding increase in the number of student placements [4–6]. The COVID-19 pandemic has made the situation worse, putting more strain on healthcare systems and leading to discussions on how to increase placement capacity [4, 7–10].

Simulation-based learning (SBL) activities are becoming increasingly popular in healthcare professional training. Several studies suggest that simulation placements can increase the capacity for practical placements [9, 11–13]. According to Gaba (2004) [14], simulation is a technique rather than a technology. It enables individuals to replace or enhance real-life experiences with guided experiences that replicate significant aspects of the real world in a fully interactive way. SBL in medicine and health provides students with interaction with real equipment or simulated patient interactions within an interactive simulated environment [15, 16].

A Delphi Consensus Study involving 79 nursing and allied health professionals reported that simulation placements could replace 11–30% of traditional practice placements [17]. Healthcare professionals use simulation placements widely to improve confidence, communication, clinical reasoning, and teamwork skills [9, 18, 19]. However, there is limited research on the immediate and long-term effects of these placements on skills, attitudes, and performance.

In 2018, Wright et al. [20] conducted an 18-day immersive simulation-based placement for 60 undergraduate pre-registration physiotherapy students in Australia. The placement involved students working six days in each of the physiotherapy core areas (cardiopulmonary, musculoskeletal, neurological) in which they interacted with professional role-play actors who acted as patients. At the beginning and end of each 6-day working, students filled out questionnaires, and the overall results showed a 35% significant improvement in the participants’ confidence in working in a clinical environment [20]. However, the benefits might be limited in the long term when students go out on subsequent placements, considering the simulated nature of the patients and the predictability of the settings, which may not mimic real-world physiotherapy practice environments. A mixed method study of 79 penultimate undergraduate physiotherapy students who participated in a 9-week peer simulation programme reported that participants found the experience valuable in helping them to identify their knowledge and skill deficits and was considered a safe and supportive environment even though students identified some lack of authenticity [21]. While the studies by Wright et al. [20] and Dalwood et al. [21] highlighted the benefit of simulation placement, they both used simulation placement as an add-on to the academic activities within the university to support student transition from university-based education to working in practice environments. Therefore, they may not have fully provided the authenticity of practice environments. Blackford et al. [18] replaced the first week of a traditional 5-week practice placement for a two-year Graduate Entry Masters physiotherapy course at an Australian university with a simulation placement using trained actors and compared it with a control group that participated in the traditional placement [18]. Both groups performed equally well at the end of the placement, but the simulated group reported feeling more confident before the start of the placement. This highlights the possibility of replacing traditional placement with simulation placement to achieve the intended learning objective.

As far as we know, this is the first study in the UK where students participate in a Physiotherapy programme with a simulation placement as their first practice placement, integrated into the course development. During this four-week-long simulated placement, students are supported by a qualified physiotherapist and exposed to real-world physiotherapy issues. The simulation placement is assessed using the Common Practice Assessment Form-CPAF- [3] and counts towards the student’s overall practice hours. This provides an authentic practice environment for the students to gain experience and interact with service users who have ongoing physiotherapy issues.

As the number of physiotherapy students rises and the healthcare industry faces more pressure, it is hoped that adding simulation placements to traditional physiotherapy programmes can improve students’ learning and expand placement opportunities. This study aimed to assess how our simulation-based placement impacts students’ knowledge, attitude, and performance when they undertake practice placements with external providers. Knowledge refers to the information, facts, theories, principles, and concepts that physiotherapy students acquire through their academic studies and practical training [22–24]. In comparison, attitude is the disposition, mindset, and emotional outlook towards their profession, patients, colleagues, and their own personal and professional growth [23, 25, 26]. Performance, on the other hand, is the practical application of knowledge and skills in a clinical setting [22, 24]. Performance also encompasses communication skills, professionalism, clinical reasoning, and ethical decision-making. High-quality performance is essential for delivering safe and effective care to patients while adhering to professional standards and guidelines. In the context of physiotherapy pre-registration students in the UK, “knowledge,” “attitude,” and “performance” are key domains in the Health and Care Professions Council’s (HCPC) Standards of Proficiency. These domains are crucial for the overall development of the students and ensure competency as future physiotherapists.

## Methods

### Simulation placement setting

In this study, the simulated patient interactions were performed by paid service users and student models who read from scripts provided by the physiotherapy practice placement team. The placement facilitator pre-selected the models.

Students were exposed to cardiorespiratory, musculoskeletal, and neurological areas of physiotherapy. The simulation setting mimicked an acute hospital, outpatient clinic, and community environment. The whole placement took place over a period of 4 weeks, 3 times a week, from 9 am to 5 pm. The first day was an induction session covering moving and handling and infection control. From day two onwards, students worked in groups of four to five participants, and they were expected to cover five stations a day, with each student leading a station. The student lead role involved timekeeping, taking patient history, leading the peer discussions on assessment and treatment planning, writing patient notes, and appointing a scribe for group notes. Each station has a service user with the relevant condition or a model with a written script that they act out. The students spent an average of 60 min per station. At the end of each day, students have a 60-minute debrief session with a practice educator, who is an HCPC registered physiotherapist. The educator observes the student’s work throughout the day. The debrief consists of constructive feedback on assessment, treatment planning, and communication with peers and service users or models. This feedback was modelled on Pendleton’s rules [27], which provide a framework for constructive feedback from the facilitator that encourages self-reflection and active participation from the learner.

Typically, the students spent five hours with patients, one hour with the practice educator, and another hour with their peers for group discussion and peer learning. There is a 30-minute lunch break and two 15-minute breaks in the mid-morning and mid-afternoon.

### Research design

This study was an exploratory qualitative research design [28] using a semi-structured interview to evaluate the impact of simulation-based practice placement within the university on the knowledge, attitude and performance of physiotherapy students during their practice placement outside the university. A semi-structured interview allows researchers to collect data from key informants about their thoughts and feelings about a particular topic [29, 30]. The consolidated criteria for reporting qualitative research (COREQ) was used for this study [31].

### Participants recruitment

Recruitment was based on a purposive sampling strategy to yield insights and in-depth understanding from the target group [32]. Eighteen Year 2 students of a 2-year MSc pre-registration physiotherapy programme in the UK who have participated in the 4-week simulation placements explained above and have completed all their practice placements with external providers, were eligible for admission into the study. The students who had interrupted their studies or had not completed all their practice placements were excluded from the study.

Students were notified of the study via a face-to-face session at one of their placement debrief sessions. Afterwards, they were approached via email with one prompt email if the candidate had not responded.

### Ethical considerations

The study was approved by the University’s Research Ethics Committee (UEP2023May01). The study was conducted in accordance with the “Code of Ethics of the World Medical Association” (Declaration of Helsinki) (1964) [33]. If participants consented to be part of the study, they were given the participants’ information sheet outlining the study aims and objectives and were asked to sign and return the consent form. Participants involved in the study were informed about their right to withdraw from the study up to two weeks after their interview. They were made aware that participation or non-participation would not affect any aspects of their learning. To ensure confidentiality, any identifiable information about participants, such as names and locations, was removed from the interview transcripts. In addition, numbered identifiers were randomly assigned to each of the participants (e.g. P1, stands for participant 1) to protect the anonymity of the participants.

### Data collection

The researchers developed the interview schedule based on published work in related field. One of the authors (YD) piloted the semi-structured interview format and questions with a colleague who was not familiar with the interviewee or the topic. This helped identify any potential leading elements in the questions and allowed the researchers to collect open-ended data to explore participants’ thoughts and feelings about their experience of simulation placement. All the interviews were conducted virtually in a 1:1 setting and recorded. The interviews lasted an average of 45 min. The researchers have extensive backgrounds in physiotherapy and higher education. All interviews were conducted by one researcher (KH) who was not involved in the cohort teaching for the academic year. The interviewer had not taught the final-year students for approximately 18 months. This was to ensure that their interactions did not influence the study’s interpretation or the participants. This contributed to the study’s impartiality.

The interview questions (see Supplementary File 1) were used as a guide to explore participants’ views on the impact of their internally conducted simulation-based placement on their subsequent practice placement with external providers. The data were collected from May to July 2023.

### Data analysis

An inductive thematic analysis based on Braun and Clarke’s approach [34] was utilised in this study. All interviews were recorded via Microsoft TEAMS and were then professionally transcribed. Transcripts were sent to participants for review. Two researchers independently used the NVivo 14 software for qualitative analysis to analyse the data. Summaries of the overall codes, references, and the initial themes were shared among the research team to explore different perspectives. Following a series of discussions within the research team, two of the researchers returned to the transcript to ensure that the themes accurately represented the data and aligned with the research objective. Once confirmed the themes were defined and named.

## Results

A total of 12 participants consented to participate in this study. They were aged between 20 and 29 (83%) and 30–39 (17%), with 58% identifying as female and 42% as male. The ethnic backgrounds of the participants were as follows: Black African (8%), Black British Caribbean (8%), Other White Background (8%), and White British (76%). Among the participants, 58% had clinical experience working in the healthcare field, holding roles such as a rehabilitation assistant, a healthcare assistant, a respiratory support worker, an occupational therapist, two osteopaths, and a sports rehabilitator. The remaining 42% had no clinical experience beyond their sports-related bachelor’s degree.

**Fig. 1:**
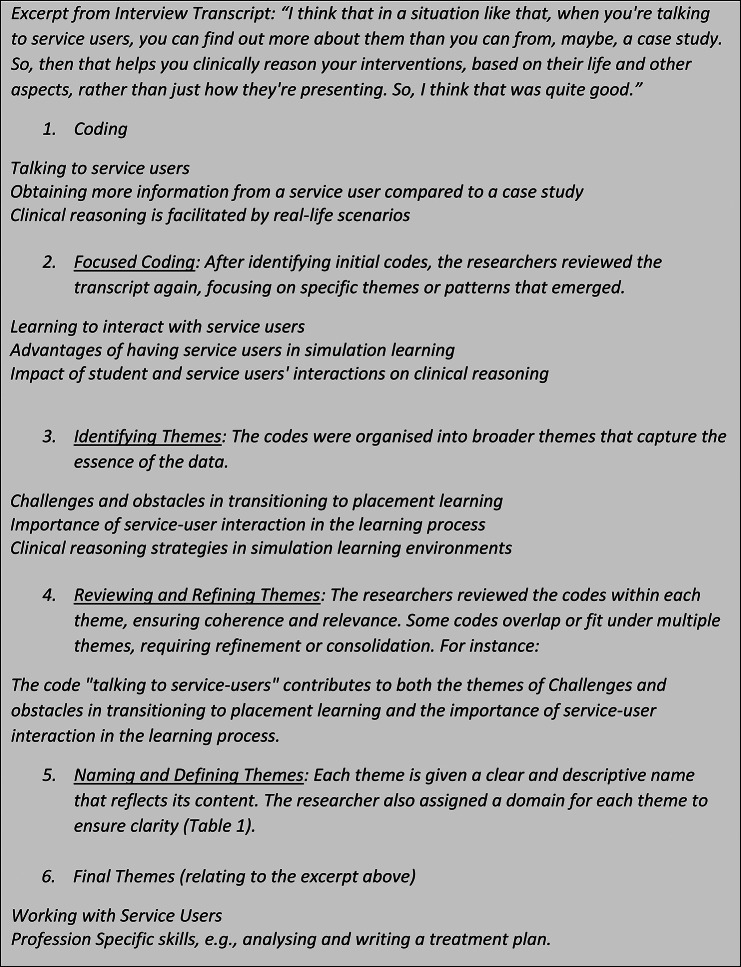
Illustrates the process of moving from transcripts to codes to themes in the data analysis

From the data, an inductive approach was employed that generated fifty-six (56) codes that were categorised into five [5] themes; [1] Working together, [2] Working with Service Users, [3] Professionalism, [4] Profession-specific practical skills and [5] Generic practical skills. An example of the process of transcribing codes to themes is presented in Fig. 1. These themes alongside their characteristics and how they map to knowledge, attitude, and performance, are described in Table 1.

**Table 1:** Theme summary table denoting K – knowledge, A – attitude, P – performance

Theme	Characteristic	Topic
(1) *Working together*	*The impact that group-based working had on encouraging shared learning and improving the ability to seek support as well as improving clinical reasoning skills and confidence.*	K
(2) *Working with Service Users*	*Analysis of the improvements in communication, confidence, and clinical reasoning skills when working with service users.*	A, P
(3) *Professionalism*	*Evaluates time management and prioritisation, adherence to code of conduct, understanding role boundaries, and personal development.*	K, A, P
(4) *Profession Specific skills*	*Specific skills are explored including subjective and objective assessment skills, intervention prescription, note-taking, and clinical reasoning.*	K, P
(5) *Generic practical skills*	*Generic skills including manual handling and use of equipment.*	K, P

### Working together

In this theme, participants shared their perspectives on how the simulation placement influenced their ability to collaborate with others. They noted that group-based work during the simulation placement enhanced their teamwork skills, aiding them in their placements when collaborating with various members of the multidisciplinary team (MDT) including occupational therapists, doctors, and nurses.

Participants unanimously expressed a positive view of working in groups. One participant described it as “fantastic.” They believed that the exposure to team-based work during the simulation placement made it easier for them to seek support when needed and assist other team members as required when on practice placement.

“*…obviously, when you go out on placement, you’re always working with someone if you have a double-hander or something. So just making sure that… When we were in our team, we would have a lead, but the others would chip in if they felt they needed to, or they had something to add. So, I think, having that team environment definitely translated well out into placement.” (P1)*

Given the diverse clinical and non-clinical backgrounds of the participants, some suggested changing groups periodically during the four-week placement would enrich their learning experience. *“I just wonder if we could have mixed the groups up just weekly. Not every day obviously, but just maybe each week we had a slightly different group so that you could learn off different people within your cohort”* (P9). Other participants felt that smaller group sizes would be a real-world representation of practice environment; *“We’re in groups of four or five and obviously typically you go in either as a single or a double. You don’t tend to go in as four or five physios to one patient” (P7).*

### Working with service users

Interacting with service users improved the simulation placement by allowing participants to practice transferable skills such as communication and clinical reasoning, which are essential for their practice placements. Participants reported increased confidence in communicating with service users, as they were unfamiliar to them, requiring them to think on their feet more readily. Assessing service users provided the opportunity to gather more detailed findings and practice maintaining a patient-focused approach during subjective history-taking, akin to real clinical scenarios.

*“I think that in a situation like that, when you’re talking to service users, you can find out more about them than you can from, maybe, a case study. So, then that helps you clinically reason your interventions, based on their life and other aspects, rather than just how they’re presenting. So, I think that was quite good.”* (P5).

Objective assessments of service users provided a more accurate representation of their condition, as seen with manual muscle testing and a timed walk test. *“The placement allowed you to really focus on things, what they mentioned. Shortness of breath, for instance, you instantly knew when you got someone up, be aware of how far you’re going to take them. If there’s sort of an area to pause if you need to around.” (*P2).

Participants agreed that more service users were neededto promote larger developments in the transferable skills mentioned above; *“then we got some service users and that was good. The more real they are, they better” (P4).*

### Professionalism

In this theme, participants highlighted the professional skills gained from their simulation placement, including understanding time management, the physiotherapist’s role in practice, adherence to the code of conduct, and personal development. *“I felt like the simulation placement kind of gave us standards as well for the placement or just, kind of, refreshed what’s expected of us, which is a good thing” (P9).* Participants commented that being exposed to a hospital environment within the simulation suites, working a nine-to-five routine and travelling to a venue that was not their usual place of study helped add to the realness of the simulation placement and better prepared them for subsequent practice placement.

*“Just because on the placement, the simulation placement, we’re wearing the uniform and we’re talking to each other in a team, we’re thinking about different scenarios, problem-solving. So then just because we’ve done that, we’ve had a little practice on that, it gives you a little more confidence going into the real world.”* (P3).

Understanding how to use the allocated time for each station and prioritise elements of assessment and intervention was commented on most readily by participants with no clinical experience. The participants reported that their confidence had improved over time during their simulation placement, evidenced by the suggestion that time to complete assessment, treatment, and note writing could have been reduced as time went on to better replicate the demands placed on them during their practice placement.

*“And then maybe time management, because the stations were timed. So maybe, kind of, just being aware that you can’t spend three hours with a patient, because you could easily do that, just being aware that you have a certain amount of time and you need to fit this, this, and this in. You need to prioritise what you need to get across and then.”* (P1).

Reflective practice formed part of the simulation placement, providing a chance to reflect on their own and others’ actions and learn from a clinical scenario they were actively involved in. There was an appreciation for conducting reflective writing and gaining feedback, as this was a fundamental element encountered on practice placement.

*“We were expected to do reflections. We were expected to be critical of what we were doing and of each other, as well as colleagues, which is something that we weren’t necessarily that used to in our own teaching before that point because we’re very much of the opinion we’d support each other through but we wouldn’t be, necessarily, critical of each other”* (P8).

One area of improvement in relation to personal development through reflective practice was the completion of the CPAF document. Participants felt simulation placement built their confidence and ability to educate their Practice Educator on how to complete this form. However, they felt that on simulation placement working with their assigned educator on a 1:1 basis would enrich the experience, thus improving their ability to respond to feedback.

### Profession specific skills

*“Your basic SOAP stuff, doing a subjective assessment, then the objective, then analysis and coming up with a plan. I found that sort of helped, definitely, towards my first placement.” (P11)*.

Enhancing subjective and objective assessment skills within all disciplines of physiotherapy was a primary goal for the students engaged in the simulation placement and received the highest amount of coding under all themes. An increased confidence gained by exposure to a variety of clinical scenarios improved “flow” from the repetition of completing subjective assessments which was considered a “softer skill” from an experienced student in the cohort. Participants were encouraged to use outcome measures when conducting objective assessments across all disciplines which received positive comments in relation to their use on subsequent placements; *“Some of the assessment skills, in simulation, we were encouraged to do outcome measures and quite a few outcome measures and to practice those with our practice patients. And I do feel like I took that into clinical placement with me.” (P9)*.

One of the participants, who, despite their clinical experience, had never been exposed to gathering past medical history of a patient, reported that conducting detailed assessments improved their active listening skills. This exposure translated into improvements in clinical reasoning by encouraging them to pay attention to the details, analysing their findings in greater depth to create their differential diagnosis.

*“Other parts of the assessment I gained from was definitely the link between different patterns, like their pain, their onset. Where it happened, when it happened. Just their general thoughts on what happened. Previous injuries before that which may link to it. I gained a lot of those types of skills in that placement.”* (P2).

Intervention was an area of contention amongst the participants, with some comments that they didn’t gain additional treatment intervention skills within the field of cardiorespiratory care. There were comments that the practice of assessment took precedence, and thus, there was limited exposure to follow-up treatment sessions. The participants with prior clinical experience also highlighted that there was a lack of feedback from the practice educator at the time of prescribing treatment to their service user or model. This limitation will be explored in the discussion. However, the ability to share knowledge and collaborate with their peers when creating treatment plans and refresh their MSK prescribing skills improved their confidence on practice placement.

*“Just because it got more ideas in my head while I was on [simulation] placement. So, I remember in my first placement, the MSK one, I remember something that happened with someone on the wards, he [fellow student] taught me extra techniques [during simulation] for treating hamstrings. And that helped me on placement, so working with peers in your group definitely helped.” (P3)*.

The diversity of note taking encountered on practice placement *was* a common theme highlighted by the participants. Students felt that the variety of software and acceptable abbreviations encountered amongst physiotherapists and the MDT would have been problematic to replicate within a simulation placement. However, building the foundations of writing SOAP notes then transferring and adjusting those skills to meet the demands of each placement was described as ‘great’ and ‘helpful’. Those students without prior clinical experience commented on the evolution of their notes throughout their placement year having gained and understood the basic concept whilst on simulation placement. The students with clinical experience commented on the lack of feedback given on the notes they sent to their educator which will be discussed within the limitations.

“*I think it’s quite difficult because I’ve noticed, from placement to placement, each educator expects you to write them differently. So, there’s only so much you can really be prepared for, but I think that the opportunity to write a set of notes for each, for one of the people we saw each day, was good, and we did have… We had an hour to do it, which you don’t necessarily have that long in placement, especially like on an MSK placement. You’ve got, like, 10 minutes to quickly write them, but I think it was good to have the experience of doing that.”* (P5).

### Generic skills

This theme will discuss generic skills that the participants felt that the simulation placement introduced them to. Generic skills such as manual handling, first aid training and infection control formed part of the induction day. Manual handling training was received with astounding positivity being described as “fantastic”, a “strong component” and a key take away for use in practice placement especially within the field of neurology. The feedback from students was encouraging in relation to the exposure to equipment found within a hospital setting such as slide sheets, banana boards and the hoist but there were requests to use the equipment more frequently throughout the four-week placement block.

*“It helped me because my first placement was MS placement, so a neurological one. It was very helpful because even at the introduction my supervisor asked me what I could do and she asked me to actually show some of our skills, and we could do it, thanks to simulation placement.”* (P6).

Students who had prior clinical experience “refreshed” previous skills during these sessions and were exposed to new methods of transferring patients, as well as prescribing and teaching patients to use walking aids, that subsequently resulted in positive patient feedback when on practice placement;

*“Then the moving and handling, actually, yes. We did a lot of moving and handling practice in the placement and it just really helped to make sure the patient felt safe when I was on placement. I had comments saying, “I feel supported when you’re moving my leg,” and whatnot, so it was good.”* (P2).

## Discussion

SBL has gained substantial traction in allied health education programmes, primarily due to the escalating scarcity of suitable clinical learning environments on both national and international levels. Existing evidence overwhelmingly supports the integration of SBL into pre-registration healthcare programmes. However, a critical gap in the literature lies in the exploration of how a simulation-based placement influences physiotherapy students as they transition to subsequent placements with external providers.

The results of this study shed light on the profound impact of a four-week simulation-based placement on the knowledge, attitude, and performance of physiotherapy students during their subsequent practice placements with external providers. To the best of our knowledge, this programme represents a unique offering in physiotherapy higher education within the UK, providing this form of simulation-based practice placement that was embedded into the course structure from its developmental phase. The primary findings of this study indicate that the simulation placement significantly enhanced students’ teamwork skills, boosted their confidence and communication abilities, and aided in the development of foundational professionalism.

Serving as an introduction to practice placement, simulation-based placement equips students with the confidence and comfort necessary within a clinical environment. It acts as a bridge between academic studies and clinical practice, exposing students to the professional roles and competencies outlined by the Chartered Society of Physiotherapy (CSP) [35] and the HCPC [36]. The results of this study show that SBL had a positive impact on improving the confidence of physiotherapy students in communication, assessment, and note-taking skills. These findings are consistent with several previous studies. For example, Wright et al. (2018) [20] found that an 18-day SBL placement improved the confidence of physiotherapy students in a similar way. In another study, Johnston et al. (2018) [37] replaced one week of a four-week placement block with simulation learning, which led to significant improvements in confidence among second-year physiotherapy students. However, completion of the subsequent three-week traditional placement did not yield significant results, highlighting the complexities of clinical practice that cannot be fully reflected in SBL. Additional research by Ohtake et al [38] found improvements in physiotherapy students’ confidence in technical, behavioural, and cognitive performance in a critical care simulation patient. Blackstock et al. (2015) [39], showed that SBL did not compromise students’ ability to achieve competency but instead showcased improved communication, assessment, management, and confidence among students.

Incorporating service user patients in healthcare education has been a practice since the 1960s [40], enhancing the student experience by allowing them to refine communication skills [39, 41, 42], build confidence [20, 37, 38], and enhance assessment abilities [18, 39, 43]. The results of this study affirm that students perceived the authenticity of their simulation placement to be enhanced through the involvement of service user patients, consistent with existing research. Striking a balance between service user and case study approaches is recommended based on this research.

The diversity in clinical backgrounds among participating students in the simulation placement was observed to enrich knowledge when working in groups. Unlike traditional placements, simulation placements expose students to a broader range of case scenarios across physiotherapy disciplines. These scenarios, each with a distinct pathophysiology, were meticulously developed by experienced clinicians and the practice placement team, ensuring exposure to a wide variety of common pathologies encountered during practice placements [20, 39]. The study’s consensus underscored the benefits of SBL on teamwork, highlighting improvements in collaboration, the ability to seek support when needed, and effective teamwork, aligning with the findings of Blackford et al. [18].

Modern healthcare and care services are increasingly adopting an integrated MDT approach to enhance care efficiency for patients and service users [44]. Producing graduates capable of effective team collaboration is a key competency highlighted by the HCPC [36]and CSP [35]. Section five of the common placement assessment form (CPAF) also emphasises the ability to work with others, reinforcing the importance of teamwork in contemporary healthcare practice. Future SBLs should consider several changes to enhance the learning process and provide a better experience for students. These changes include changing groups throughout the placement, reducing group sizes, and shortening the time to complete assessments and treatments as the placement progresses. Additionally, collaborating with other healthcare students can also be beneficial.

While the study identified several positive aspects of the simulation placement, areas for improvement were also acknowledged. Specifically, enhancing feedback mechanisms. Although Pendleton’s approach to feedback seems to have worked well, students desired more feedback from the facilitators’ views. It is likely that the practice educator will require training and practice to provide constructive feedback and enable self-reflection effectively. The completion of the CPAF in a one-to-one with the practice educator and ensuring the availability of necessary equipment were also noted as necessary improvements. In similar findings to Wright et al [20], we found that SBL has limitations in its ability to replicate elements of practice such as treatment intervention. Participants also felt that the scarcity of certain equipment impacted their placement performance, particularly in manual handling scenarios. Addressing these equipment challenges, integrating intervention-related scenarios, and refining feedback mechanisms are essential to further enhance simulation-based placements’ effectiveness in physiotherapy education.

## Conclusion

SBL, when used as a comprehensive placement, has the potential to provide a valuable educational experience for physiotherapy students. It can help better equip students for the constantly evolving healthcare environment and enhance their knowledge and skills. According to this study, SBL has a positive impact on knowledge, attitude, and performance, highlighting its potential to prepare students for successful integration into the healthcare environment. The findings suggest that the integration of SBL into physiotherapy education should continue, with a focus on refining its structure and feedback mechanisms to optimise educational benefits. However, caution should be exercised in implementing any changes to ensure that the educational benefits are maximised.

To enhance and improve the authenticity of SBL, our recommendations include recruiting more service users, incorporating and encouraging more intervention-based elements, and increasing the contact time between the practice educator and students. Future research should aim to further validate and expand on these findings, exploring the long-term retention and application of knowledge acquired through SBL in real clinical scenarios.

### Electronic supplementary material

Below is the link to the electronic supplementary material.

Supplementary Material 1

## Data Availability

The datasets generated during and/or analysed during the current study are available from the corresponding author upon request.

## Abbreviations

A: Attitude
CSP: Chartered Society of Physiotherapy
CPAF: Common Practice Assessment Form-CPAF
COREQ: Consolidated criteria for reporting qualitative research
HCPC: Health and Care Professions Council
K: Knowledge
MDT: Multidisciplinary team
P: Performance
SBL: Simulation-based learning
UK: United Kingdom
