# Supplementary material for: The impact of simulation-based learning on the knowledge, attitude and performance of physiotherapy students on practice placement
Source: BMC Med Educ. 2024 Jul 22;24:786. doi: 10.1186/s12909-024-05718-2 (PMC11264462; doi:10.1186/s12909-024-05718-2)
Supplement: Supplementary file 1 — Supplementary Material 1 [file 12909_2024_5718_MOESM1_ESM.docx]

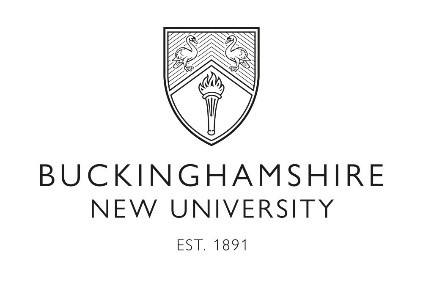


**The impact of simulation-based learning on the knowledge, attitude and performance of physiotherapy students on practice placement**

**Interview Questions**

Duration: Approximately 30-45 minutes

Correlation between student prior experience and the benefit of simulation placement

1. **When you first heard about the simulation placement, what were your immediate thoughts and feelings?**

- What are your feelings now? Have they changed?

1. **Prior to your simulation placement, did you have any clinical experience?**

- If so, tell us about that experience.

1. **Describe the assessment skills you felt you gained during simulation placement and tell me how you used some of those skills on practice placement.**

Prompt:

- Were there any gaps in your knowledge when you went out on practice placement?
- Include subjective and objective assessment
- Do you believe that the clinical competencies such as manual handling, first aid training and infection control helped you when you went out on your practice placement? What can be improved if not?

1. **Note taking forms a legal part of our role as a physiotherapist – how do you feel the simulation placement aided in preparing you for writing clinical notes on your practice placements?**

Prompt:

- Is there anything, having completed your clinical placement that you wish you had been able to practice during the simulation?

1. **Did completing the simulation placement impact your ability to prescribe treatment for patients on practice placement for each of the domains of physiotherapy (cardiorespiratory, neurological and musculoskeletal)?**

1. **Are there any other skills you felt you gained from the simulation placement?**

Prompt:

- Clinical reasoning skills, diary management, manual handling, communication, timekeeping, professionalism.

1. **In relation to the assessment of the simulation placement, do you feel that the use of the CPAF document was useful for future placements?**

1. **Overall, do you feel that completing the simulation placement impacted your performance on your practice placement with external providers?**

1. **Having completed your 1000 hours of placement and be nearing graduation, is there anything you would add or change to the running of the simulation placement to help students when they go out on practice placement?**

*Thank you very much for taking part in this study, your involvement is extremely valuable to shaping the curriculum for future physiotherapist in higher education here at BNU and in the wider communities.*
